# Supplementary material for: Saturation of radiative heat transfer due to many-body thermalization
Source: Sci Rep. 2020 Jun 2;10:8938. doi: 10.1038/s41598-020-65555-3 (PMC7265501; doi:10.1038/s41598-020-65555-3)
Supplement: Supplementary file 1 — Supplementary Information. [file 41598_2020_65555_MOESM1_ESM.pdf]

Supplementary Information

Saturation of radiative heat transfer due to many-body thermalization

Ivan Latella[1,2,*], Riccardo Messina[2], Svend-Age Biehs[3], J. Miguel Rubi[1,4], and Philippe Ben-Abdallah[2,*]

[1]Departament de Física de la Matèria Condensada, Universitat de Barcelona, Martí i Franquès 1, 08028 Barcelona, Spain
[2]Laboratoire Charles Fabry, UMR 8501, Institut d'Optique, CNRS, Université Paris-Saclay, 2 Avenue Augustin Fresnel, 91127 Palaiseau Cedex, France
[3]Institut für Physik, Carl von Ossietzky Universität, D-26111 Oldenburg, Germany
[4]PoreLab–Center of Excellence, Norwegian University of Science and Technology, N-7491 Trondheim, Norway
*ilatella@ub.edu;pba@institutoptique.fr

Correlation matrices of the substrate and bath fields

Here we obtain some expressions used in the main text. In particular, we derive the correlation matrix of the substrate field given in Eq. (17) and an explicit expression for the matrix $\mathbb{B}(\omega)$ defining the correlations of the bath field in Eq. (18). Below we also give explicit expressions for the factors $|\xi_i(\omega)|^2$ appearing in the exchanged powers (20) and (21). We start by considering the correlations of the substrate field.

Taking into account that the substrate field at the point $\mathbf{r}$ propagates to the right, we expand it as

$$\mathbf{E}^1(\omega) = \sum_p \int \frac{d^2\mathbf{k}}{(2\pi)^2} \exp(i\mathbf{K}\cdot\mathbf{r})\hat{\boldsymbol{\epsilon}}^+(\mathbf{k},\omega,p)\mathcal{E}(\mathbf{k},\omega,p), \tag{1}$$

where $\mathbf{K} = (\mathbf{k}, k_z)$ is the wave vector for which the component parallel to the surface is $\mathbf{k} = (k_x, k_y)$ with $k = |\mathbf{k}|$. Here,

$$\hat{\boldsymbol{\epsilon}}^\pm(\mathbf{k},\omega,\mathrm{TE}) = \frac{1}{k}(-k_y, k_x, 0), \qquad \hat{\boldsymbol{\epsilon}}^\pm(\mathbf{k},\omega,\mathrm{TM}) = \frac{c}{\omega k}(\pm k_x k_z, \pm k_y k_z, -k^2) \tag{2}$$

are the unit polarization vectors and $\mathcal{E}$ is the associated field mode. The correlation function of these field modes is given by[1,2]

$$\langle \mathcal{E}(\mathbf{k},\omega,p)\mathcal{E}^*(\mathbf{k}',\omega',p')\rangle = (2\pi)^2\delta(\mathbf{k}-\mathbf{k}')\delta(\omega-\omega')\delta_{pp'}\frac{\pi\hbar\omega^2}{\varepsilon_0 c^2} n_1(\omega)\frac{1}{k_z}\left[\Pi^{\mathrm{pw}}\left(1-|r_1^p|^2\right) + \Pi^{\mathrm{ew}} 2i\mathrm{Im}\left(r_1^p\right)\right]. \tag{3}$$

Taking into account this correlation function and using cylindrical coordinates in which $d^2\mathbf{k} = k dk d\phi$, the components of the correlation matrix of the substrate field can be written as

$$\begin{aligned}\langle E_i^1(\omega)E_j^{1*}(\omega')\rangle = {}& \frac{4\pi\hbar\omega^2}{\varepsilon_0 c^2} n_1(\omega)\delta(\omega-\omega') \\ & \times \sum_p \int_0^\infty \frac{dk}{8\pi} k \frac{1}{k_z}\left[\Pi^{\mathrm{pw}}\left(1-|r_1^p|^2\right)\int_0^{2\pi}\frac{d\phi}{2\pi}\hat{\epsilon}_i^+\hat{\epsilon}_j^+ + \Pi^{\mathrm{ew}} 2i\mathrm{Im}\left(r_1^p\right) e^{i2k_z(d+R)}\int_0^{2\pi}\frac{d\phi}{2\pi}\hat{\epsilon}_i^+\hat{\epsilon}_j^-\right],\end{aligned} \tag{4}$$

where we have made use of the properties of the polarization vectors in such a way that $\Pi^{\mathrm{pw}}\hat{\epsilon}_i^+\hat{\epsilon}_j^{+*} = \Pi^{\mathrm{pw}}\hat{\epsilon}_i^+\hat{\epsilon}_j^+$ and $\Pi^{\mathrm{ew}}\hat{\epsilon}_i^+\hat{\epsilon}_j^{+*} = \Pi^{\mathrm{ew}}\hat{\epsilon}_i^+\hat{\epsilon}_j^-$. Performing the angular integral in the above equation leads to the substrate filed correlation matrix (17) of the main text, which is proportional to the matrix $\mathbb{S}$ defined by

$$\mathbb{S}(\omega) = \int_0^\infty \frac{dk}{8\pi} k \begin{pmatrix} f & 0 & 0 \\ 0 & f & 0 \\ 0 & 0 & g \end{pmatrix}, \tag{5}$$

with

$$f = \Pi^{\mathrm{pw}}\frac{1}{2k_z}\left[\left(1-|r_1^{\mathrm{TE}}|^2\right) + \frac{c^2k_z^2}{\omega^2}\left(1-|r_1^{\mathrm{TM}}|^2\right)\right] + \Pi^{\mathrm{ew}}\frac{i}{k_z}\left[\mathrm{Im}\left(r_1^{\mathrm{TE}}\right) - \frac{c^2k_z^2}{\omega^2}\mathrm{Im}\left(r_1^{\mathrm{TM}}\right)\right]e^{i2k_z(d+R)}, \tag{6}$$

$$g = \Pi^{\mathrm{pw}}\frac{c^2k^2}{\omega^2 k_z}\left(1-|r_1^{\mathrm{TM}}|^2\right) + \Pi^{\mathrm{ew}}\frac{i2c^2k^2}{\omega^2 k_z}\mathrm{Im}\left(r_1^{\mathrm{TM}}\right)e^{i2k_z(d+R)}. \tag{7}$$

Furthermore, for convenience, we now express the imaginary part of the vacuum Green's function in the coincidence limit as

$$\mathrm{Im}\mathbb{G}^{(0)}(\omega)=\int_0^\infty \frac{dk}{8\pi}k\begin{pmatrix}q&0&0\\0&q&0\\0&0&s\end{pmatrix},\qquad q=\Pi^{\mathrm{pw}}\frac{1}{k_z}\left(1+\frac{c^2k_z^2}{\omega^2}\right),\qquad s=\Pi^{\mathrm{pw}}\frac{2c^2k^2}{\omega^2k_z}. \tag{8}$$

This expression is obtained by writing the vacuum Green's function in terms of the polarization vectors in cylindrical coordinates and integrating over the angular variable. Besides, the imaginary part of the scattering Green's function in the coincident limit takes the form

$$\mathrm{Im}\mathbb{G}^{(\mathrm{R})}(\omega)=\int_0^\infty \frac{dk}{8\pi}k\begin{pmatrix}t&0&0\\0&t&0\\0&0&u\end{pmatrix}, \tag{9}$$

where

$$t=\Pi^{\mathrm{pw}}\frac{1}{k_z}\mathrm{Re}\left[\left(r_1^{\mathrm{TE}}-\frac{c^2}{\omega^2}k_z^2r_1^{\mathrm{TM}}\right)e^{i2k_z(d+R)}\right]+\Pi^{\mathrm{ew}}\frac{i}{k_z}\left[\mathrm{Im}(r_1^{\mathrm{TE}})-\frac{c^2k_z^2}{\omega^2}\mathrm{Im}(r_1^{\mathrm{TM}})\right]e^{i2k_z(d+R)}, \tag{10}$$

$$u=\Pi^{\mathrm{pw}}\frac{2c^2k^2}{\omega^2k_z}\mathrm{Re}\left(r_1^{\mathrm{TM}}e^{i2k_z(d+R)}\right)+\Pi^{\mathrm{ew}}i\frac{2c^2k^2}{\omega^2k_z}\mathrm{Im}(r_1^{\mathrm{TM}})e^{i2k_z(d+R)}, \tag{11}$$

so that the imaginary part of the total Green's function $\mathrm{Im}\mathbb{G}(\omega)=\mathrm{Im}\mathbb{G}^{(0)}(\omega)+\mathrm{Im}\mathbb{G}^{(\mathrm{R})}(\omega)$ can be readily decomposed into propagating and evanescent wave contributions as well.

Taking into account that the correlations of the bath field are given by $\mathbb{B}(\omega)=\mathrm{Im}\mathbb{G}(\omega)-\mathbb{S}(\omega)$, this matrix can be written as

$$\mathbb{B}(\omega)=\int_0^\infty \frac{dk}{8\pi}k\begin{pmatrix}v&0&0\\0&v&0\\0&0&w\end{pmatrix}, \tag{12}$$

where $v=q+t-f$ and $w=s+u-g$. Working out these coefficients we obtain

$$v=\Pi^{\mathrm{pw}}\frac{1}{2k_z}\left[\left|1+r_1^{\mathrm{TE}}e^{i2k_z(d+R)}\right|^2+\frac{c^2k_z^2}{\omega^2}\left|1-r_1^{\mathrm{TM}}e^{i2k_z(d+R)}\right|^2\right],\qquad w=\Pi^{\mathrm{pw}}\frac{c^2k^2}{\omega^2k_z}\left|1+r_1^{\mathrm{TM}}e^{i2k_z(d+R)}\right|^2, \tag{13}$$

where we observe that there is no contribution from evanescent waves.

Finally, we give an explicit expression for the factor $|\xi_i(\omega)|^2$ appearing in the spectral power, where the quantity $\xi_i(\omega)$ has been introduced in Eqs. (13) of the main text. Using the relation $|\zeta|^2=\mathrm{Re}^2(\zeta)+\mathrm{Im}^2(\zeta)$ and taking into account the expression of the scattering Green's function given in Eq. (11) of the main text, we get

$$|\xi_x|^{-2}=\left[1+\int_0^\infty\frac{dk}{8\pi}k\frac{\omega^2}{c^2}\mathrm{Im}(\alpha a)\right]^2+\left[\int_0^\infty\frac{dk}{8\pi}k\frac{\omega^2}{c^2}\mathrm{Re}(\alpha a)\right]^2, \tag{14}$$

with $\xi_y=\xi_x$, and $|\xi_z|^{-2}$ can be obtained from the above equation by replacing $a\to b$, the coefficients a and b being given in Eqs. (11) in the main text as well.

References

1. Messina, R. & Antezza, M. Scattering-matrix approach to Casimir-Lifshitz force and heat transfer out of thermal equilibrium between arbitrary bodies. *Phys. Rev. A* **84,** 042102 (2011).

2. Latella, I., Ben-Abdallah, P., Biehs, S.-A., Antezza M. & Messina, R. Radiative heat transfer and nonequilibrium Casimir-Lifshitz force in many-body systems with planar geometry. *Phys. Rev. B* **95,** 205404 (2017).
